# Dataset on blood biomarkers and GRACE score measured at admission for myocardial infarction in a large secondary hospital

**DOI:** 10.1016/j.dib.2018.09.126

**Published:** 2018-10-03

**Authors:** Victor J. van den Berg, Majorie van Toorenburg, Olivier Drexhage, Eric Boersma, Isabella Kardys, Victor A.W.M. Umans

**Affiliations:** a Northwest Clinics, Alkmaar, the Netherlands; b Erasmus MC, Rotterdam, the Netherlands

## Abstract

The GRACE score is currently the most widely used model to assess patient prognosis after myocardial infarction (MI). We have demonstrated that the prognostic performance of the GRACE score can be improved by adding blood biomarkers measured routinely at hospital admission in our study recently published in the International Journal of Cardiology: “Addition of routinely measured blood biomarkers significantly improves GRACE risk stratification in patients with myocardial infarction”.

In this Data-in-Brief article we present additional original data from our dataset. This dataset consists of clinical and biomarker information and follow-up data of 2055 confirmed MI patients. In 143 of these patients the endpoint (all-cause mortality or reMI) occurred during six months follow-up. We describe the differences in baseline characteristics between ST-elevation MI (STEMI) patients and non-STEMI patients, differences in biomarker levels at admission between patients in whom the endpoint occurred and patients who remained endpoint-free, and associations of the biomarkers with the endpoint. Moreover, we show additional statistical results of analyses that compare the original GRACE-only model with our extended GRACE/biomarker model.

**Specifications table**

**Table:** 

Subject area	*Clinical cardiology*
More specific subject area	*Myocardial infarction, acute coronary syndrome*
Type of data	*Table, figures*
How data was acquired	*From a prospective database consisting of all patients treated for myocardial infarction in the Northwest Clinics, the Netherlands, between 2013 and 2016. Missing data were completed and clinical outcomes were added retrospectively.*
Data format	*Filtered and analyzed*
Experimental factors	*N/A*
Experimental features	*Summary statistics and AUCs for different biomarkers*
Data source location	*Alkmaar, the Netherlands*
Data accessibility	*Data is with this article and not in a public repository*

**Value of the data**

• Data from our cohort recently published in the related research article shows that the ability of the GRACE score for detecting MI patients at high risk for mortality or MI within 6 months, can be significantly improved by adding several biomarkers measured routinely at admission.
• Our large cohort provides insights into current prognosis after treatment for myocardial infarctions.
• This data-in-brief shows the differences in clinical characteristics between patients with ST-elevation myocardial infarction and patients with myocardial infarction without ST-elevation.
• The data shows associations for many common biomarkers and clinical outcomes in patients with myocardial infarctions. In addition, corresponding AUCs for each biomarker are provided.
• Subgroup analyses demonstrate that biomarkers are incremental to GRACE in all examined subsets of patients.

## 1 Data

The data shared is generated from a large prospective dataset containing all consecutive hospitalizations for myocardial infarctions (MI) between 2013 and 2016 from the Northwest Clinics. In this data, we have correlated patient characteristics and biomarker data with clinical outcomes. Here we show the details from our statistical analysis that resulted in an improved prognostic model for MI patients.

– Table 1: Baseline characteristics of ST-elevation MI (STEMI) patients and non-STEMI patients separately.

**Table 1:** Baseline characteristics for myocardial infarction patients with or without ST-elevation.

	**NSTEMI (*N* = 1078)**	**STEMI (*N *= 977)**	***p*-value**
**Age**	68.8 (12.6)	65.3 (12.8)	<0.001
**Male gender, n (%)**	708 (65.7)	687 (70.3)	0.028
**Risk factors, n (%)**			
** None**	111 (11.0)	118 (12.7)	0.277
** Hypertension**	541 (53.5)	360 (38.7)	<0.001
** Hypercholesterolemia**	344 (34.0)	248 (26.6)	<0.001
** Family history of CAD**	400 (39.6)	377 (40.5)	0.710
** Diabetes, IDDM**	76 (7.5)	28 (3.0)	<0.001
** Diabetes, NIDDM**	121 (12.0)	78 (8.4)	0.011
** Current smoker**	322 (31.8)	417 (44.8)	<0.001
**Cardiovascular disease history, n (%)**			
** MI**	204 (25.0)	92 (12.1)	<0.001
** PCI**	190 (23.4)	80 (10.5)	<0.001
** CABG**	71 (8.7)	19 (2.5)	<0.001
** Stroke/TIA**	54 (6.6)	23 (3.0)	0.001
**Admission infarct**			
** Highest troponin I, ug/L**	2.05 (0.39, 7.35)	24.54 (6.75, 68.17)	<0.001
** Highest CK, U/L**	170 (92, 401)	803 (382, 2079)	<0.001
** Days in hospital**	3 (2., 4)	2 (2, 3)	<0.001
**GRACE and individual components**			
** GRACE risk score**	141 (116, 165)	170 (146, 195)	<0.001
** Systolic blood pressure, mmHg**	145 (25)	127 (24)	<0.001
** Heart rate, beats/min**	70 (63, 83)	71 (63, 80)	0.968
** Creatinin, mol/L**	84 (72, 101)	80 (69, 94)	<0.001
** Killip class I,% (N)**	991 (91.9)	911 (93.2)	0.007
** ST deviation**	250 (23.2)	977 (100.0)	<0.001
** Elevated cardiac enzymes**	865 (80.2)	722 (73.9)	0.001
** Cardiac arrest**	13 (1.2)	60 (6.1)	<0.001
**Imaging, n (%)**			
**No visible CAD**	93 (9.7)	10 (1.0)	overall
			<0.001
**1 vessel**	351 (36.7)	476 (49.7)	
**2 vessels**	237 (24.8)	261 (27.2)	
**3 vessels**	240 (25.1)	195 (20.4)	
**Left main**	36 (3.8)	16 (1.7)	
**Percutaneous intervention, % (n)**			
** Left main**	24 (3.4)	11 (1.2)	0.005
** Left anterior descending artery**	309 (43.8)	397 (43.9)	1.000
** Left circumflex artery**	200 (28.4)	144 (15.9)	<0.001
** Right coronary artery**	209 (29.6)	369 (40.8)	<0.001

*a* = mean (standard deviation).

*b* = median (25th, 75th percentile).

*c* = measured every six hours during admission until the value starts to lower.

Categorical variables for cases and non-cases were compared using chi-square or Fisher׳s exact test, whichever was appropriate. Differences for continuous variables in the same groups were tested using the student t-test for normally distributed variables and Mann-Whitney test for non-normally distributed variables.
– Table 2: Average biomarker values in the entire cohort, in patients in whom the endpoint occurred and in endpoint-free patients.

**Table 2:** Median biomarker levels.

**Biomarker, unit**	**Reference values**	**Median values (IQR)**	**No event, *n* = 1912**	**Event, n = 143**	***P*-value**
**Troponin I, ug/L**	(0.00, 0.04)	0.15 (0.05, 0.76)	0.14 (0.05, 0.69)	0.34 (0.07, 1.63)	<0.001
**Creatine kinase, U/L**	(0, 171)	124 (78, 210)	124 (80, 209.25)	110 (65, 215)	0.068
**C-reactive protein, mg/L**	(0, 5)	2.8 (1.2, 6.5)	2.6 (1.2, 6.0)	6.6 (2.7, 24.0)	<0.001
**ASAT, U/L**	(0, 35)	25 (18, 37)	24 (18, 36)	25 (18, 43)	0.348
**ALAT, U/L**	(0, 45)	23 (17, 32)	23 (17, 32)	22 (16, 30)	0.225
**Alkalic phosphatase, U/L**	(0, 120)	72 (60, 87)	72 (60, 87)	77 (65, 97)	<0.001
**Gamma-glutamyltransferase, U/L**	(0, 55)	28 (19, 43)	27 (19, 42)	31 (23, 51)	0.009
**Cholesterol, mmol/L**	(0.0, 6.4)	5.1 (4.3, 6.0)	5.2 (4.3, 6.0)	4.6 (3.5, 5.6)	<0.001
**Triglycerides, mmol/L**	(0.6, 2.2)	1.3 (0.8, 2.0)	1.3 (0.8, 2.0)	1.2 (0.9, 1.9)	0.814
**HDL cholesterol, mmol/L**	(0.9, 1.7)	1.2 (1.0, 1.4)	1.2 (1.0, 1.4)	1.1 (1.0, 1.4)	0.309
**LDL cholesterol, mmol/L**	(1.5, 4.5)	3.3 (2.4, 4.1)	3.3 (2.5, 4.1)	2.6 (1.8, 3.7)	<0.001
**Sodium, mmol/L**	(134, 145)	137 (136, 139)	137 (136, 139)	137 (135, 139)	0.001
**Potassium, mmol/L**	(3.4, 4.9)	3.9 (3.6, 4.2)	3.9 (3.6, 4.1)	4.0 (3.8, 4.5)	<0.001
**Glucose, mmol/L**	(3.3, 7.8)	7.8 (6.5, 9.6)	7.8 (6.5, 9.5)	8.3 (6.8, 11.2)	0.002
**Urea, mmol/L**	(2.0, 8.0)	6.0 (4.9, 7.5)	5.9 (4.8, 7.3)	8.0 (6.0, 11.0)	<0.001
**Hemoglobin, mmol/L**	(8.5, 11.0)^a^	8.7 (8.1, 9.3)	8.8 (8.1, 9.3)	8.1 (7.3, 8.8)	<0.001
**Thrombocytes, 10^9/L**	(150, 400)	237 (200, 284)	238 (201, 285)	230 (194, 278)	0.097
**Leukocytes, 10^9/L**	(4.0, 10.0)	9.3 (7.4, 11.8)	9.3 (7.3, 11.8)	9.6 (7.5, 12.1)	0.386

LDL: low-density lipoprotein; HDL: high-density lipoprotein; ASAT: aspartate aminotransferase; ALAT: alanine aminotransferase;

U/L: units per liter; Mmol/L: millimol per liter; ug/L: microgram per liter; mg/L: milligram per liter.

a Reference value for males, for females the reference value is 7.5–10.0 mmol/L.
– Table 3: Associations between patients characteristics, biomarkers and clinical outcome at six months. We also added the corresponding AUCs.

**Table 3:** Associations between clinical characteristics and biomarkers measured at admission and clinical outcomes during 6 months of follow-up.

	**Entire cohort *n* = 2055 (143 events)**		**STEMI patients *n *= 977 (58 events)**		**NSTEMI patients *n* = 1078 (85 events)**	
	*OR (95%CI)*	*AUC*	*OR (95%CI)*	*AUC*	*OR (95%CI)*	*AUC*
***Clinical variables, unit of increase***						
Systolic blood pressure, 10 mm Hg	0.96 (0.89, 1.02)	0.54	0.98 (0.87, 1.09)	0.55	0.90 (0.82, 0.99)	0.57
Heart rate, 10 bpm	1.26 (1.16, 1.37)	0.61	1.37 (1.19, 1.57)	0.62	1.19 (1.06, 1.33)	0.60
Killip class, class	3.33 (2.47, 4.50)	0.61	3.17 (2.15, 4.72)	0.60	3.66 (2.32, 5.72)	0.61
Elevated cardiac enzymes, yes	1.58 (1.02, 2.56)	0.54	1.54 (0.82, 3.18)	0.54	1.54 (0.85, 3.04)	0.53
Cardiac arrest, yes	0.56 (0.14, 1.54)	0.51	0.53 (0.09, 1.76)	0.51	0.97 (0.05, 5.03)	0.50
***Biomarkers measured at admission, unit of increase***						
Troponin I, 100 ug/L	1.14 (0.36, 2.00)	0.59	1.25 (0.43, 2.20)	0.62	1.15 (1.08, 1.24)	0.56
Creatine kinase, 100 U/L	0.97 (0.91, 1.02)	0.55	1.02 (0.96, 1.07)	0.51	0.82 (0.67, 0.96)	0.58
C-reactive protein, 10 mg/L	1.14 (1.09, 1.19)	0.69	1.13 (1.07, 1.20)	0.72	1.16 (1.08, 1.24)	0.66
ASAT, 100 U/L	1.17 (0.92, 1.42)	0.52	1.34 (1.05, 1.66)	0.61	0.59 (0.20, 1.18)	0.51
ALAT, 100 U/L	1.14 (0.77, 1.50)	0.53	1.28 (0.94, 1.80)	0.57	0.07 (0.01, 0.52)	0.60
Alkalic phosphatase, 100 U/L	2.93 (1.84, 4.75)	0.59	2.76 (1.28, 6.53)	0.59	2.96 (1.66, 5.37)	0.59
Gamma-glutamyltransferase, 100 U/L	1.16 (0.91, 1.42)	0.57	1.64 (1.07, 2.39)	0.61	1.03 (0.63, 1.32)	0.54
Cholesterol, 1 mmol/L	0.71 (0.61, 0.82)	0.62	0.62 (0.48, 0.80)	0.63	0.77 (0.64, 0.92)	0.60
Triglycerides, 1 mmol/L	0.99 (0.82, 1.19)	0.51	0.94 (0.66, 1.29)	0.52	0.95 (0.74, 1.20)	0.50
HDL cholesterol, 1 mmol/L	0.96 (0.55, 1.63)	0.53	1.16 (0.46, 2.76)	0.51	0.82 (0.41, 1.60)	0.55
LDL cholesterol, 1 mmol/L	0.68 (0.58, 0.80)	0.63	0.58 (0.44, 0.76)	0.65	0.77 (0.63, 0.93)	0.60
Sodium, 1 mmol/L	0.89 (0.84, 0.94)	0.58	0.88 (0.81, 0.96)	0.60	0.88 (0.82, 0.94)	0.59
Potassium, 1 mmol/L	2.37 (1.73, 3.25)	0.62	3.18 (1.83, 5.47)	0.62	1.99 (1.33, 2.98)	0.61
Glucose, 1 mmol/L	1.12 (1.07, 1.16)	0.58	1.16 (1.09, 1.24)	0.60	1.10 (1.04, 1.16)	0.58
Urea, 1 mmol/L	1.26 (1.21, 1.32)	0.71	1.31 (1.21, 1.42)	0.67	1.24 (1.18, 1.31)	0.73
Creatinin, 10 mg/mmol	1.13 (1.10, 1.18)	0.69	1.27 (1.18, 1.37)	0.69	1.09 (1.05, 1.14)	0.69
Hemoglobin, 1 mmol/L	0.48 (0.40, 0.57)	0.68	0.55 (0.42, 0.72)	0.65	0.44 (0.35, 0.55)	0.71
Thrombocytes, 10^11/L	0.92 (0.71, 1.15)	0.54	1.27 (0.90, 1.72)	0.51	0.71 (0.49, 0.99)	0.57
Leukocytes, 10^9/L	1.01 (0.97, 1.04)	0.52	1.03 (0.96, 1.10)	0.53	1.01 (0.96, 1.05)	0.54

**Fig. 1:**
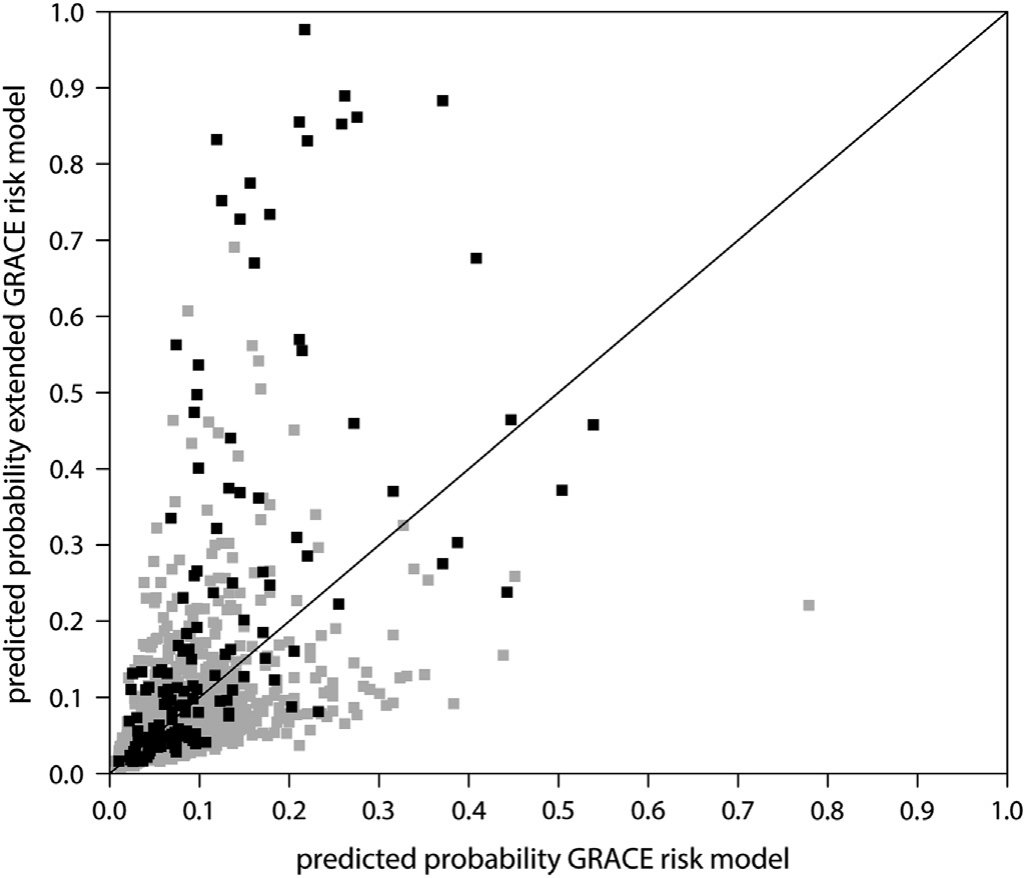
Predicted risks. Grey squares are patients without an event in 6 months after hospitalization for MI Black squares are patients with an event in 6 months after hospitalization for MI.
– Table 4: Comparison of the performance of the GRACE-only model and GRACE model extended with available biomarker data. The performance is compared in the entire cohort and in different subgroups of the cohort.

LDL: low-density lipoprotein; HDL: high-density lipoprotein; ASAT: aspartate aminotransferase; ALAT: alanine aminotransferase; *n* = number; OR: odds ratio; 95%CI: 95% confidence interval; U/L: units per liter; Mmol/L: millimol per liter; ug/L: microgram per liter; mg/L: milligram per liter; STEMI: ST-elevation myocardial infarction; NSTEMI: non ST-elevation myocardial infarction.
– Fig. 1 Depicts the differences in estimated risk from the GRACE-only model and the GRACE model extended with available biomarker data.

**Table 4:** Performance of GRACE-only model and full model in different subcategories.

Cohort	No. of patients	No. of events	Area under ROC-curve	
			GRACE-only model	Extended GRACE model
Full cohort	2055	143	0.70	0.76
STEMI	977	58	0.71	0.74
NSTEMI	1078	85	0.75	0.79
Male	1395	86	0.66	0.72
Female	660	57	0.74	0.82
Age 65 or lower	906	24	0.59	0.61
Age above 65	1149	119	0.65	0.74

No.: number; STEMI: ST-elevation myocardial infarction; NSTEMI: Non ST-elevation myocardial infarction; Extended GRACE model: model containing the GRACE risk score and admission levels of the following blood biomarkers: urea, sodium, potassium, alkaline phosphatase, LDL cholesterol, glucose, hemoglobin and C-reactive protein.

## 2 Experimental design, materials and methods

From our prospective hospital dataset, we have retrieved the data on all hospitalizations for MI between 2013 and 2016. From this hospitalization set, we selected all unique patients that were directly sent to the Northwest Clinics for treatment and ensuing admission. Patients referred to our hospital solely for revascularization were thus excluded. In addition, we excluded patients that transferred to other hospitals outside of our region for further (outpatient) treatment before six months of follow-up. For patients that were treated for MI multiple times during 2013 and 2016, the first admission with complete blood profile was chosen as the index admission.

For all patients, we had a complete biomarker profile, consisting of 19 established biomarkers measured upon admission: Troponine I, creatine kinase, C-reactive protein, urea, creatinine, sodium, potassium, ASAT, ALAT, alkaline phosphatase, Gamma-GT, total cholesterol, high-density lipoprotein cholesterol, triglycerides, glucose, low-density lipoprotein cholesterol, leukocytes, hemoglobin and thrombocytes. Moreover, for all patients the admission-GRACE score was calculated. This score is used to calculate the risk of re-MI or all-cause mortality within six month after the index MI [1], [2].

In total, the data consist of the information of 2055 patients admitted for MI. Of these patients, 977 suffered a STEMI and 1078 a NSTEMI. Table 1 shows the differences in baseline characteristics between STEMI and NSTEMI patients. STEMI patients were on average younger, more often male, smoked more but otherwise had less risk factors than NSTEMI patients. STEMI patients also less often had a history of cardiovascular disease and a much higher GRACE score.

The endpoint occurred in 143 patients during the 6 months follow-up. In Table 2 the differences in average biomarker levels between cases and non-cases are shown. We also calculated, using logistic regression, the odds ratio per unit increase for experiencing the endpoint for all available biomarkers (Table 3). In addition, we added the odds ratios for each separate component of the GRACE-score. Finally, using the odds ratios, we calculated AUCs to be able to compare prognostic values of both the clinical characteristics and the biomarker data. An overview of the odds ratios and AUCs are shown in Table 3.

Finally, we extended the GRACE-score by adding the available biomarkers from our dataset in order to improve its prognostic value [3]. In our new extended model, the following biomarkers were included: urea, sodium, potassium, alkaline phosphatase, LDL cholesterol, glucose, hemoglobin and C-reactive protein. The distribution of the predictions for each patient for the GRACE-only model and the extended GRACE model are depicted in Fig. 1. Table 4 shows the AUCs for the two models in the total cohort as well as in different subgroups of the total cohort.
