# Supplementary material for: Dataset on blood biomarkers and GRACE score measured at admission for myocardial infarction in a large secondary hospital
Source: Data Brief. 2018 Oct 3;21:371–6. doi: 10.1016/j.dib.2018.09.126 (PMC6197650; doi:10.1016/j.dib.2018.09.126)
Supplement: Supplementary file 1 — Supplementary material [file mmc1.docx]

Conflict of Interest

All authors declare they do not have any conflict of interest.
